# Supplementary material for: DMRforPairs: identifying Differentially Methylated Regions between unique samples using array based methylation profiles
Source: BMC Bioinformatics. 2014 May 15;15:141. doi: 10.1186/1471-2105-15-141 (PMC4046028; doi:10.1186/1471-2105-15-141)
Supplement: Additional file 2 — DMRforPairs output for the comparison of A431-MCF7 and NA17018-NA17105. Please start from the HTML files in each folder. Available via the BMC Bioinformatics website. [file 1471-2105-15-141-S2.zip › 1394847754114233_MOESM2_ESM/A431_MCF7/figures/14212.pdf]

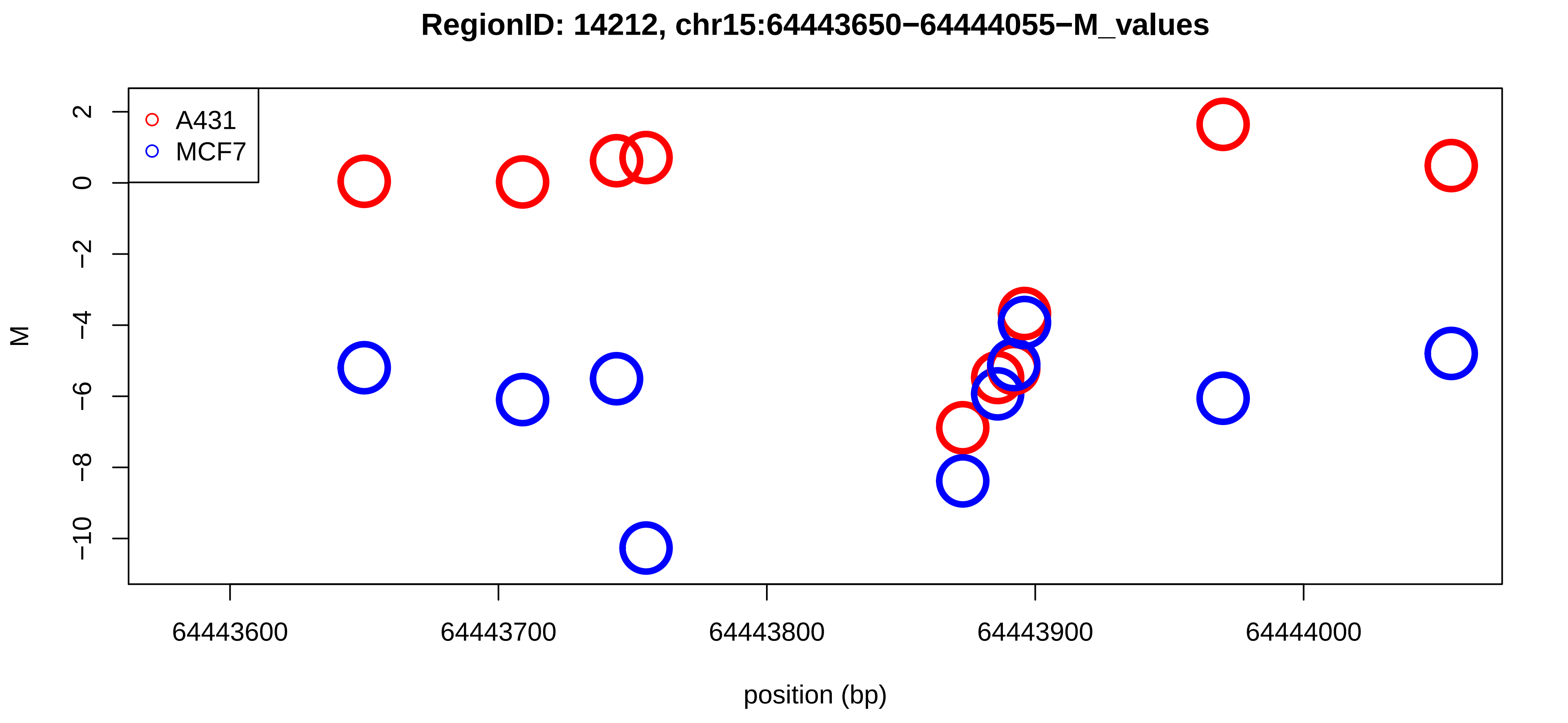

RegionID: 14212, chr15:64443650−64444055−M_values
A431
MCF7
M
2
0
−2
−4
−6
−8
−10
64443600
64443700
64443800
64443900
64444000
position (bp)

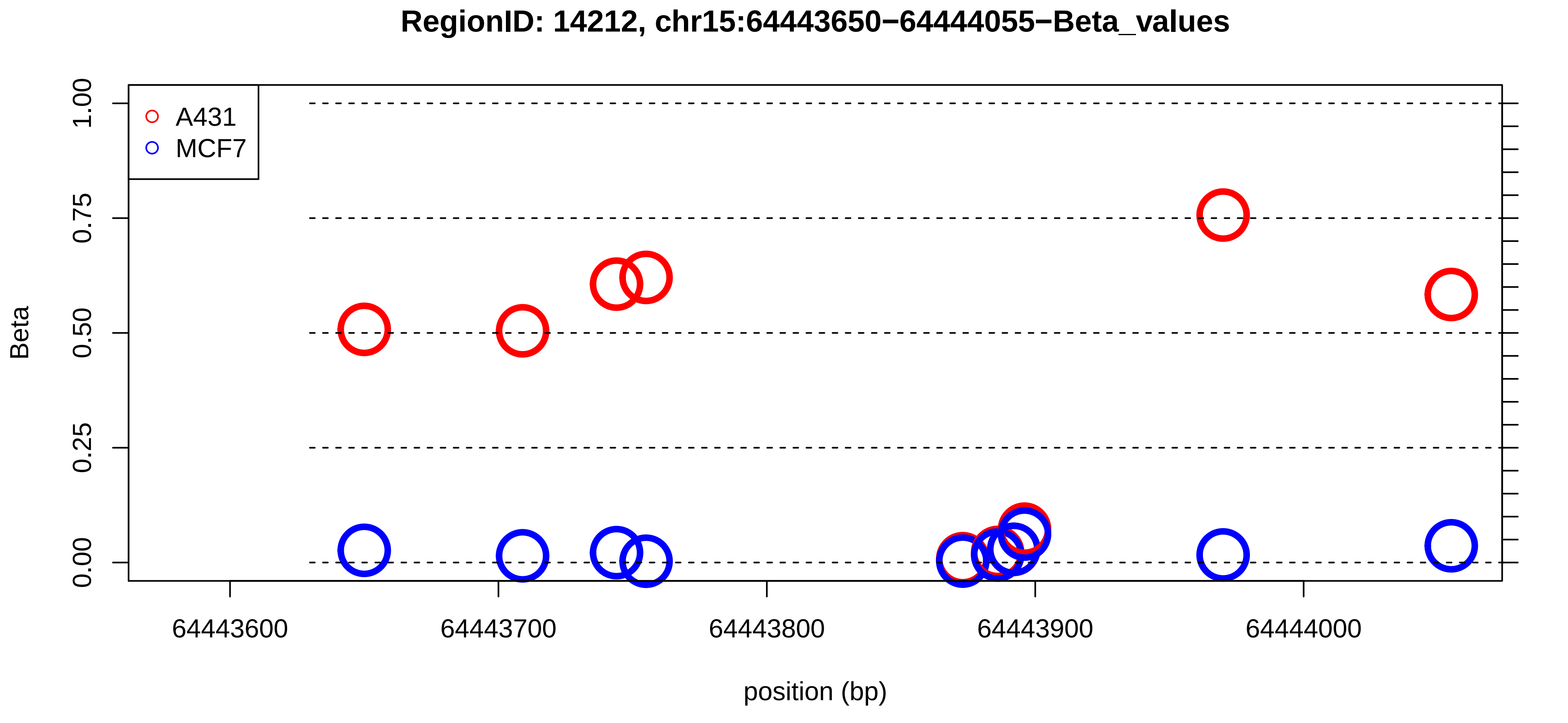

RegionID: 14212, chr15:64443650−64444055−Beta_values
A431
MCF7
Beta
1.00
0.75
0.50
0.25
0.00
64443600
64443700
64443800
64443900
64444000
position (bp)

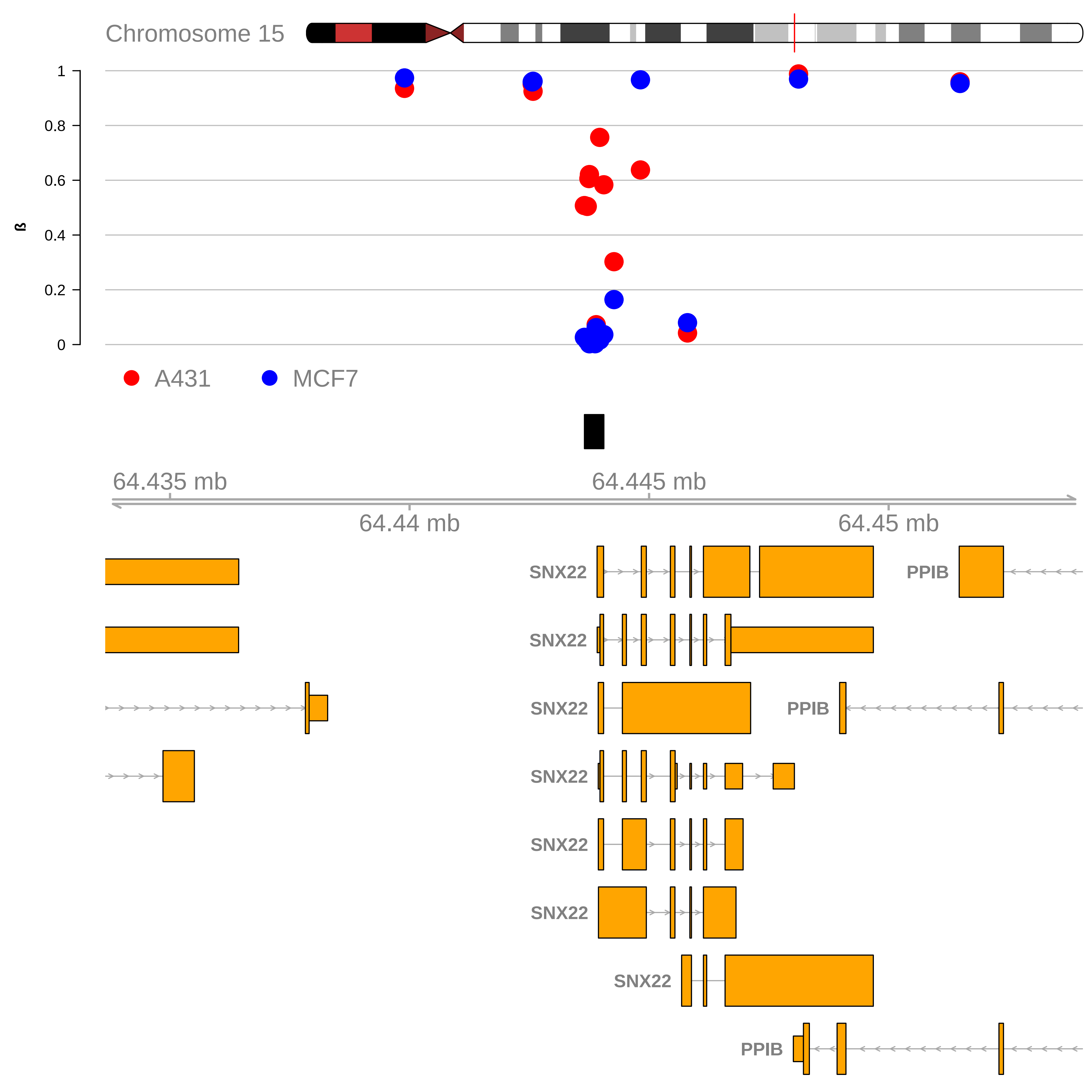

Chromosome 15
1
0.8
0.6
0.4
0.2
0
ß
A431
MCF7
64.435 mb
64.445 mb
64.44 mb
64.45 mb
SNX22
PPIB
SNX22
SNX22
PPIB
SNX22
SNX22
SNX22
SNX22
PPIB
